# Global research trends on physical education practices: a bibliometric analysis and science-mapping study

**DOI:** 10.3389/fspor.2025.1532754

**Published:** 2025-03-20

**Authors:** Vicenç Hernández-González, Josep Maria Carné-Torrent, Carme Jové-Deltell, Joaquin Reverter-Masia

**Affiliations:** ^1^ Human Movement Research Group (RGHM), University of Lleida, Lleida, Spain; ^2^ Physical Education and Sport Section, University of Lleida, Lleida, Spain

**Keywords:** physical education, practicum, bibliometrics, productivity, network analysis, web of science

## Abstract

**Background:**

Physical Education teacher training, specifically internships, require the application of theory to real-life contexts. Although these internships are mandatory in training programs, they are often undervalued. This study aims to provide an overview of research in this field and highlight future trends to contribute to the development of strategies for improving teacher training. The study analyzes the training of Physical Education teachers, emphasizing the importance of professional practice in applying theory to real-world contexts.

**Methods:**

To identify trends and improve teacher training, a bibliometric analysis was conducted on 83 publications retrieved from the Social Sciences Citation Index and the Science Citation Index Expanded in Web of Science.

**Results:**

Since 2010, a significant increase in publications on this topic has been observed, mostly in English, with 1,827 citations and an average of 22.01 citations per article. The documents had 198 authors from 27 countries, with the United States being the most prolific. The analysis revealed three research clusters: one focused on “attitudes” and “inclusion” of children with disabilities, another on “Physical Education” and “teachers’ beliefs,” and a third centered on “practices” and “perception” in adapted Physical Education. International collaboration was variable, with institutions predominantly from the United States, Brazil, and Spain. The most influential journals included Adapted Physical Activity Quarterly and Physical Education and Sport Pedagogy.

**Conclusions:**

The study reveals a notable growth in research on Physical Education practicums since 2010, with three main thematic clusters and a low level of author collaboration.

## 1 Introduction

Currently, the need for highly trained teachers is increasingly evident (1). Research on teacher training over the past decade has highlighted the complexities and systematic nature of this process, emphasizing the crucial role of key inputs, such as material resources, curriculum, faculty, and infrastructure, along with the learning environment in shaping the outcomes of future professionals (2). However, this process faces considerable challenges, including a shortage of trained personnel, outdated training programs, high costs, and the need for effective evaluation of training quality and impact (3–5).

In this context, professional internships play a fundamental role in the development of undergraduate and postgraduate students. Practical experience is essential in shaping the identity and skill set of future professionals (6, 7). This process involves multiple factors—such as the curriculum, student interns, teacher educators, and the environment—who collectively influence the quality of graduates (2, 8).

These internships allow for acquired knowledge to be applied in a real and supervised environment (9). Additionally, they help students gradually transition into professional life by providing networking opportunities, the development of interpersonal skills, employment prospects, and engagement in meaningful tasks (7, 10). Therefore, internships are considered a type of field-based learning experience and are valued by teachers, students, and educational institutions seeking to hire future professionals. These experiences can considerably influence the career trajectories of pre-service teachers (11).

In this context, experiential learning is an educational approach that emphasizes learning through direct experience, which can be important for professional development and the acquisition of future professional competencies (12). A recent meta-analysis conducted by Burch et al. (13) on experiential learning concluded that students who applied it experienced superior learning outcomes.

Within Sports Sciences, specifically Physical Education, knowledge can be applied effectively inside and outside the classroom (14). Therefore, internships have become an essential component of professional Physical Education preparation programs across various countries (2, 15–19). However, higher education has not always adequately valued practical experience, instead traditionally prioritizing academic content over practical subjects (20, 21).

Recognizing that the literature offers multiple definitions of practicums, we define them as crucial spaces for connecting academic theory with future work environments (22, 23). This period is fundamental for university students (24, 25), as it allows them to explore the internal processes of their professional development and thus fosters their identity construction (25–27). As highlighted by Lizana (25) and Mañas-Olmo (28), this topic has garnered increasing interest in the scientific community in recent years.

In this context, it is necessary to thoroughly review all available information on the research on educational and training practices in Physical Education (17). Given the volume of resources invested in this research, it is crucial to have tools that quantify their results and impact. In this respect, bibliometrics has established itself as a fundamental tool in research evaluation (29).

Bibliometrics, as a statistical analysis method, plays a key role in identifying research trends and evaluating its impact (30). It enables the analysis of large volumes of scientific literature and guides decision-making in the development of new projects (31, 32). Moreover, it facilitates the study of author cooperation, citations, journals, and institutions and thereby contributes to the visibility of research and the assessment of its reach and influence (33).

Bibliometric analyses reveal the most productive authors, collaborations among them, the most active research centers, and how the work is disseminated at national and international levels (34, 35). Understanding these dynamics helps consolidate collaborative networks, foster the exchange of ideas, and expand scientific discussions on practices in Physical Education (29, 36).

Although bibliometric mapping in educational practices has been widely studied in recent years (28, 17, 37–39), to date, no bibliometric studies have been conducted specifically on Physical Education practicums. Exploring the field's current state and future perspectives allows for a better understanding of the knowledge structure and development in the educational domain, providing new strategies for teacher training.

Therefore, this study aims to provide an overview of research in this field and to highlight future trends, with the goal of contributing to the development of strategies that improve teacher training.

## 2 Materials and methods

### 2.1 Search strategy and eligibility criteria

The raw data were obtained and downloaded from the Web of Science Core Collection (WoSCC), which includes the Science Citation Index Expanded (SCIE) and the Social Sciences Citation Index (SSCI), developed by Clarivate Analytics of Thomson Scientific. WoS hosts a vast number of high-quality, impactful scientific studies, and it is the most comprehensive and exhaustive collection of information available worldwide (33, 40). Because citation data vary by database, WoS is considered the best option for bibliometric studies (40, 41).

The search strategy employed the terms (“practicum” OR “practice”) AND (“Physical Education”), which allowed for the retrieval of all articles and reviews related to specific practices in the field of Physical Education published online between 1900 and 2024 in the WoSCC, including SCIE and SSCI.

**Figure 1:**
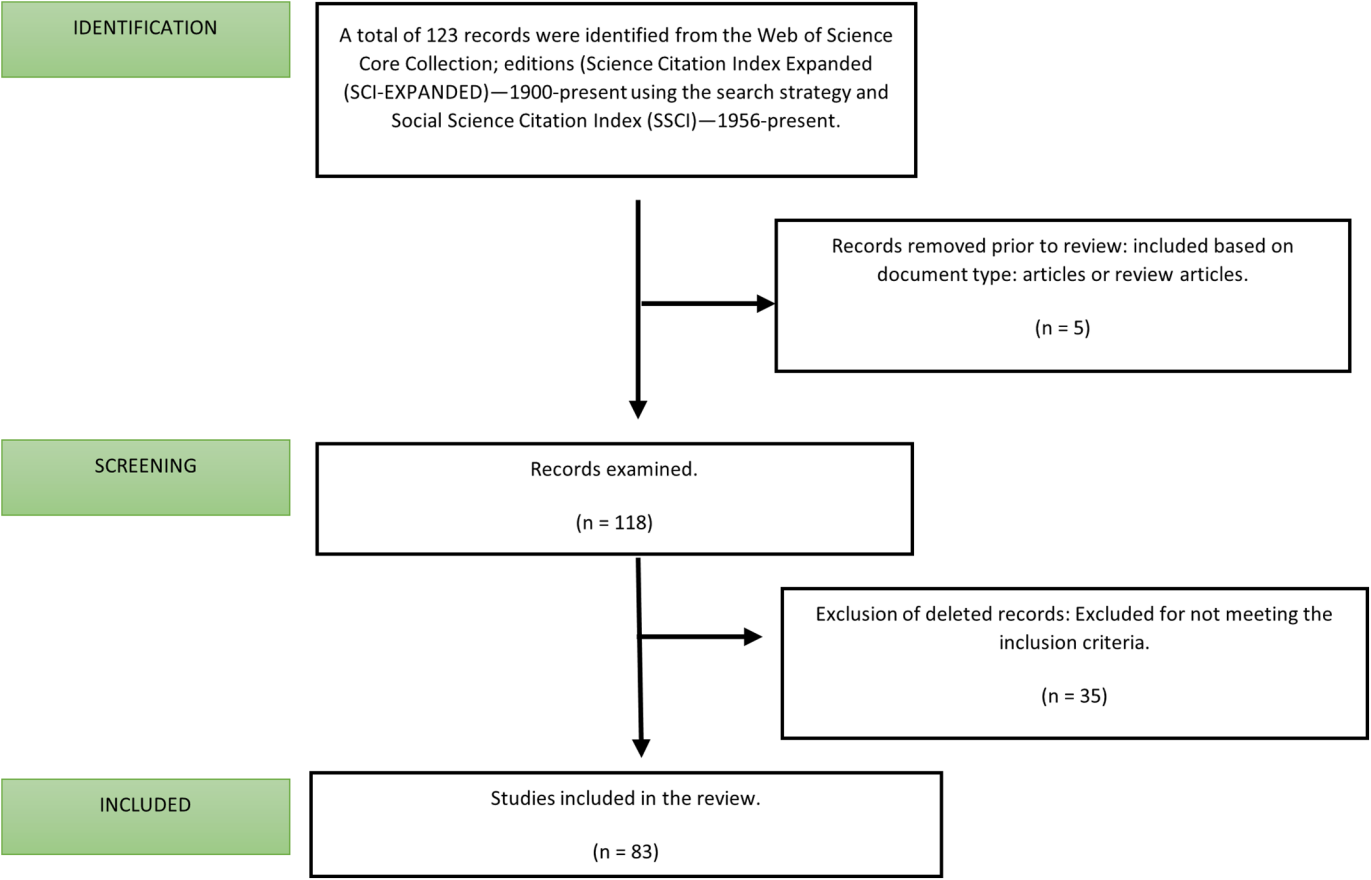
Study flowchart.

To reduce the risk of bias, two authors independently retrieved the documents on May 1, 2024, between 6:00 PM and 9:00 PM. Afterward, the documents that met the inclusion and exclusion criteria were selected and exported. The inclusion criteria were a document type of article or review; the exclusion criteria were a document type of proceeding paper or editorial material and anomalous document. The detailed search and selection process is illustrated in Figure 1.

Because only articles and reviews were considered, five documents were excluded. Additionally, 35 documents were removed after both authors reviewed them and determined they did not match the terms used in the search strategy. Ultimately, 83 original articles and reviews were included for the in-depth analysis and visualization.

The selected articles were exported to an Excel file, where the following variables were recorded: citation number, journal name, publication year, author and co-author names and surnames, total number of authors, authors' geographical locations and associated institutions, article title, document type (article or review), abstract, and corresponding author. For the authorship analysis, all individuals who contributed to each study were included in the count.

Regarding the bibliometric analysis by country, following previous studies (29, 32), the country of origin of each author involved in the study was considered as was the number of citations an article received. If a country received multiple citations for the same study, the citations were not counted more than once, even if the authors were from different institutions within that country. The total number of articles per country was determined based on whether at least one author was affiliated with that country.

To calculate each author's citation number and Hirsch index (h index), this study considered only articles and citations related to the study's subject matter.

### 2.2 Bibliometric analysis

Objective and evaluative bibliometric techniques were employed to visualize and analyze the research on Physical Education practicums. Objective bibliometrics focuses on measuring the literature's quantity and the citation numbers, and it includes a citation analysis (42). The key indicators of productivity, impact, and publication quality are the number of publications (Np), the number of citations (Nc), and the average number of citations per article (Na), respectively.

On the other hand, evaluative bibliometrics provides quantitative assessments of contributions to the research field from various countries, authors, journals, and institutions, and it uses the h index as the main metric (43). This type of analysis helps to identify the most influential articles in the field's evolution, and it detects current research hotspots and future trends (44).

### 2.3 Statistical analysis

SPSS 27.0 software (IBM, United States) was used for the correlation analysis. Additionally, Microsoft Excel was used to perform a linear regression analysis and assess the publication trend over time. A polynomial model was applied to predict the increase in the number of publications.

To identify cooperation networks and analyze keyword co-occurrence, this study used VOSviewer 1.6.18 (CWTS, Netherlands), a tool widely employed in bibliometric analyses (45). Moreover, the R Bibliometrix package (version 4.2.2) was used because the program is designed to conduct quantitative analyses in scientometrics and informetrics (46). This tool extracts metadata, thus allowing the classification and analysis of the bibliographic data imported from WoS and large volumes of research data over a specific period.

The analysis identified concurrent relationships between the keywords used and their distances on the knowledge map. Map interpretation was enhanced through the use of color, size, and distance rankings (clusters or nodes) of the evaluated keywords. Furthermore, the software generated visual representations of the knowledge. Finally, MapChart was used to create a customized map.

## 3 Results

A total of 83 documents were analyzed, of which 80 were articles and 3 were reviews, and the majority of these documents was published in the last decade. The predominant language was English (75 documents), followed by Portuguese (7) and Spanish (1). In total, the publications received 1,827 citations, with an average of 22.01 citations per article. The study involved 198 authors from 27 countries, and the articles were published in 29 journals.

### 3.1 Global publishing landscape

**Figure 2:**
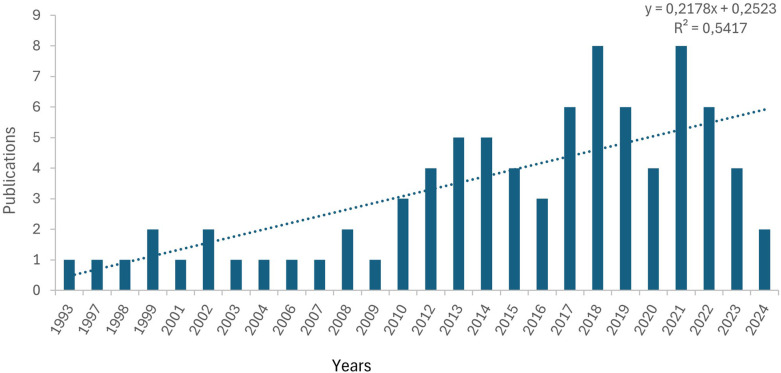
Annual article distribution pattern.

The analyzed documents were published between 1993 and 2024, with 82% appearing after 2010. Although some fluctuations were observed, the overall trend showed a clear upward trajectory. We performed a publication trend analysis using a fitting curve, which suggested a sustained upward trend in the coming years. A notable increase in the volume of publications was evident, with exponential growth in recent years. Our linear regression analysis revealed a significant, positive correlation between the number of annual publications and the publication year (*R*^2^ = 0.736, *p* < 0.001) (see Figure 2). This finding indicates that scholars are paying increasing attention to the research field of Physical Education practicums.

**Figure 3:**
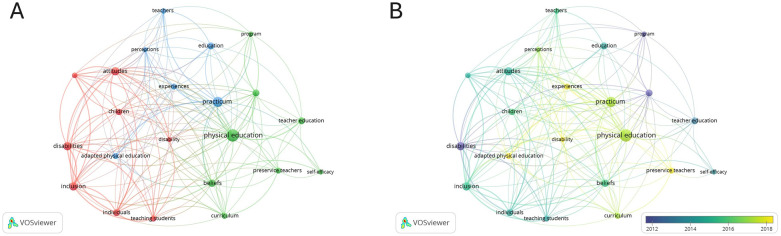
**(A)** keyword co-occurrence network. Node size indicates occurrence frequency. The curves between the nodes represent keyword co-occurrence in the same publication. Shorter node distances indicate higher keyword co-occurrences. **(B)** Relationship between keywords over time. Keyword colors indicate their average publication period, calculated by taking the average of the publication years of all publications.

A keyword analysis offers valuable insights into key research topics and emerging trends. This analysis was developed using VOSviewer and included titles, keywords, and abstracts. The network was created from 391 keywords, which were selected for further analysis if they appeared in at least five publications, resulting in 22 keywords selected for network inclusion. The results of this analysis are presented in Figure 3, which shows three distinct nodes.

The first node (red) consisted of eight keywords—including “attitudes,” “children,” “disabilities,” and “inclusion”—and “attitudes” had the greatest number of connections, so the node was thus titled. This keyword shared 18 links with the other keywords on the map and had a total link strength (TLS) of 50. In descending link order were the keywords “inclusion” (*n* = 16; TLS 51), “disability” (*n* = 16; TLS 26), “individuals” (*n* = 16; TLS 39), “disabilities” (*n* = 15; TLS 43), “children” (*n* = 15; TLS 30), “teaching students” (*n* = 15; TLS 36), and “practical experiences” (*n* = 13; TLS 29). The keywords in this node reflected a research focus on practices associated with children with disabilities and attitudes toward their inclusion. This assumption was confirmed by the fact that the highest connection strength was between the keywords “inclusion” and “attitudes.”

The second node (green) included eight keywords such as “Physical Education,” “beliefs,” “curriculum,” “student teachers,” and “teacher training.” This node was named after its most prominent keyword, “Physical Education,” which dominated the node by linking to 20 of the other keywords on the map and by having a TLS of 52. In descending link order were the keywords “beliefs” (*n* = 16; TLS 32), “curriculum” (*n* = 14; TLS 20), “student teachers” (*n* = 11; TLS 13), “teacher training” (*n* = 10; TLS 13), “programs” (*n* = 10; TLS 15), and “self-efficacy” (*n* = 5; TLS 8). The keywords in this node reflected a research focus on the beliefs held by future Physical Education teachers. This conclusion was reinforced by the strong connection between the keywords “Physical Education” and “beliefs.”

The third node (blue) included six keywords such as “practices,” “education,” “perception,” “teachers,” and “adapted Physical Education.” This node was named “practices,” the most prominent keyword with the greatest relevance, as it linked to 19 of the node's other keywords and had a TLS of 45. In descending link order were the keywords (*n* = 11; TLS 14), and “experiences” (*n* = 10; TLS 15). The keywords in this node reflected a research focus on practices as well as on teachers’ perceptions and experiences regarding adapted Physical Education. This conclusion was reinforced by the strong connection between the keywords “practices” and “perception” (see Figure 3A).

The most frequently used keywords occurred from 2012 to 2018. Older keywords were represented in blue, while more recent ones were shown in yellow. The nodes' color ranges and sizes indicated that the most recurrent keywords emerged from 2016 to 2018. The nodes with more recent keywords were related to “Physical Education,” “practices,” “adapted Physical Education,” and “student teacher.” In contrast, the node associated with “disabilities,” “programs,” “efficacy,” and “teacher training” corresponded to older keywords. These findings demonstrate that the most recent research has focused on adapted Physical Education, particularly in its relationship with teaching practices during training periods (Figure 3B).

### 3.2 Authors and Co-authors

A total of 198 authors contributed to the articles on Physical Education practicums. The number of authors per article ranged from 1 to 6, with an average of 2.95. Our analysis of the 10 most productive authors was based on the number of articles published without considering authorship position, and it revealed that S.R. Hodge, T. Sato, and G. González-Calvo were the most prolific authors in this field.

S.R. Hodge, a professor at the College of Education and Human Ecology at Ohio State University, United States, had the highest citation number (257) with six published articles and an h index of 5 in relation to Physical Education practicums. He averaged 42.83 citations per article. T. Sato—initially affiliated with the Faculty of Health and Sport Sciences at the University of Tsukuba, Japan, and later with Kent State University, School of Teaching, Learning, and Curriculum Studies in Kent, United States—was the second most productive author, with five articles that were cited 93 times for an average of 18.5 citations per article and an h index of 4. Third was G. González-Calvo, from the University of Valladolid, Spain, who published 4 articles and had an h index of 5 and an average of 53.5 citations per article (see Table 1).

**Table 1:** Top 10 authors in the research on physical education practicums.

Author	Np	H index	First author	Last author	Co-author	Nc
Hodge, S.R.	6	5	4	1	1	257
Sato, T.	5	4	4	0	1	93
González-Calvo, G.	4	5	2	1	1	214
Rossi, T.	4	3	1	3	0	69
Tinning, R.	4	4	0	1	2	104
Haegele, J.A.	4	3	0	3	1	67
Hutzler, Y.	3	3	2	0	1	153
Sirna, K.	3	3	2	0	1	103
Varea, V.	3	3	2	0	1	193

Np, number of publications; Nc, number of citations.

Our analysis showed that the total citation number was not related to the number of authors participating in the studies (*r*s = −0.092, *p* = 0.008). Furthermore, the author collaboration network analysis revealed no significant collaboration network. Some of the 27 elements in the network exhibited no connections, and the largest connected group consisted of only four members.

**Figure 4:**
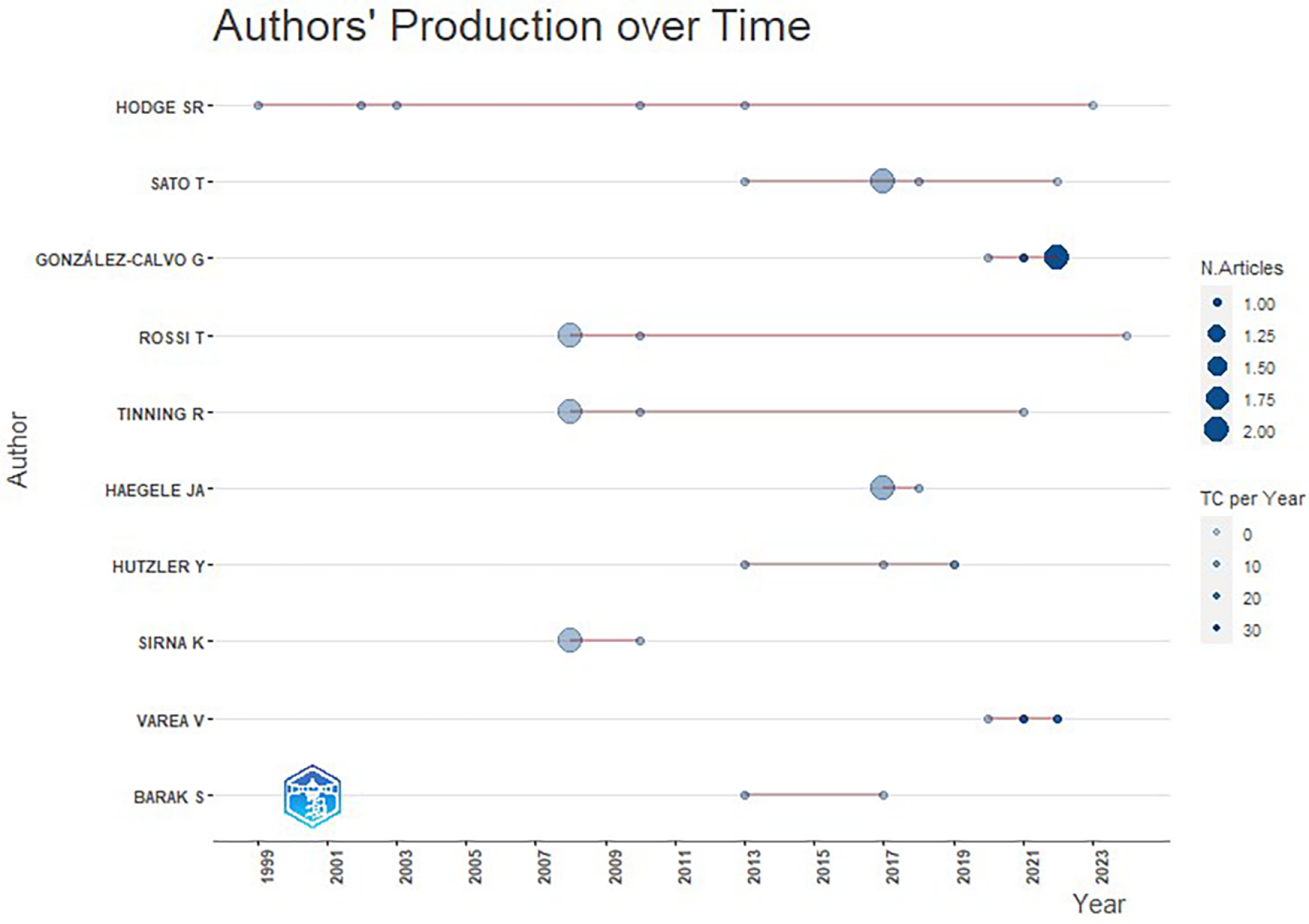
Authors’ production levels over time on physical education practicums. Circle size indicates the number of documents, and color indicates the number of citations.

The productivity of the leading authors over time can effectively reflect the temporal distribution and number of articles published during a given period. As shown in Figure 4, S.R. Hodge has been the most productive and consistent author in the research on Physical Education practicums over the past two decades, steadily and annually producing articles and having a high total citation index. In contrast, T. Sato, G. González-Calvo, and T. Rossi have published over the past decade but only in recent years have they achieved excellent performance in terms of output and total citations.

### 3.3 Countries, institutions, and collaborations

Researchers from 27 countries published 83 articles on the analyzed topic. Table 2 shows the 10 most productive countries. The United States contributed 29 documents; followed by Brazil, who contributed 9; and Australia, Canada, and Spain, who each contributed 8. The countries with the highest citation numbers were the United States, China, Ireland, and Sweden. Regarding the average number of citations per article, Israel led with 48.25; followed by Ireland, with 44; and Sweden, with 35.17.

**Table 2:** Countries with the highest citation numbers in the research on physical education practicums.

Country	Nc	Np	Na	H index
United States	711	29	24.5	17
Brazil	32	9	3.56	3
Australia	142	8	17.75	6
Canada	149	8	18.63	5
Spain	180	8	30	4
People's Republic of China	235	6	29.38	5
Sweden	211	6	35.17	4
Ireland	220	5	44	4
Norway	62	5	12.4	4
Israel	193	4	48.25	4

Np, number of publications; Nc, number of citations; Na, average number of citations per article.

**Figure 5:**
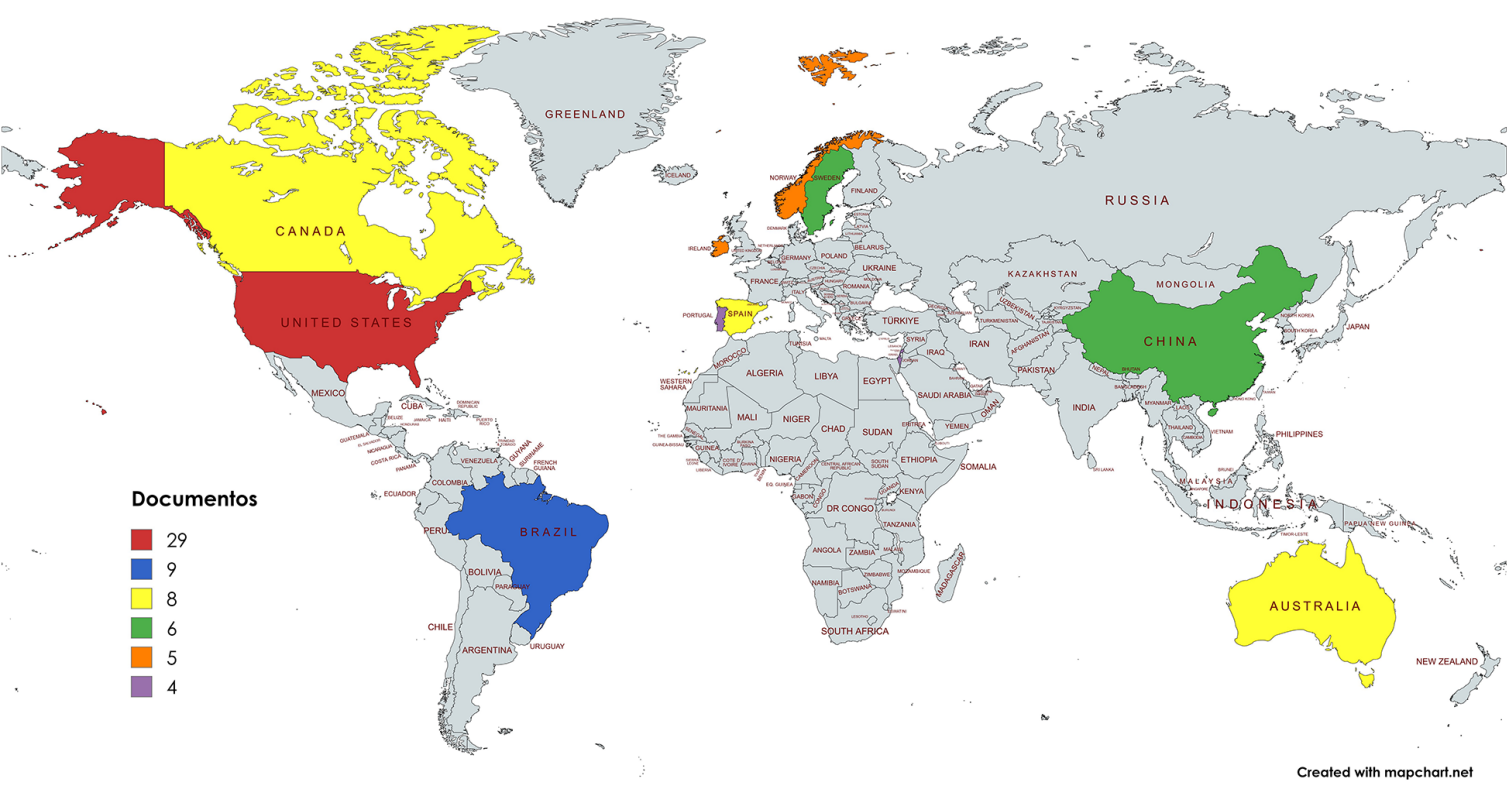
Distribution map showing the number of articles published by country (mapChart).

The publications on Physical Education practicums originated from 27 countries, distributed as follows: 17 in Europe, 4 in Asia, 3 in the Americas, 1 in Africa, and 2 in Oceania. Figure 5 illustrates the global distribution of these publications by country and region. The five most productive countries, which represented only 18.5% of the total, generated nearly 53.4% of the publications. More than half of the countries contributed only one or two documents. A single publication may be linked to multiple countries or institutions because authors can have multiple affiliations at the country and the institution levels. The United States produced the largest number of publications, followed by Brazil, Spain, Canada, and Australia.

**Figure 6:**
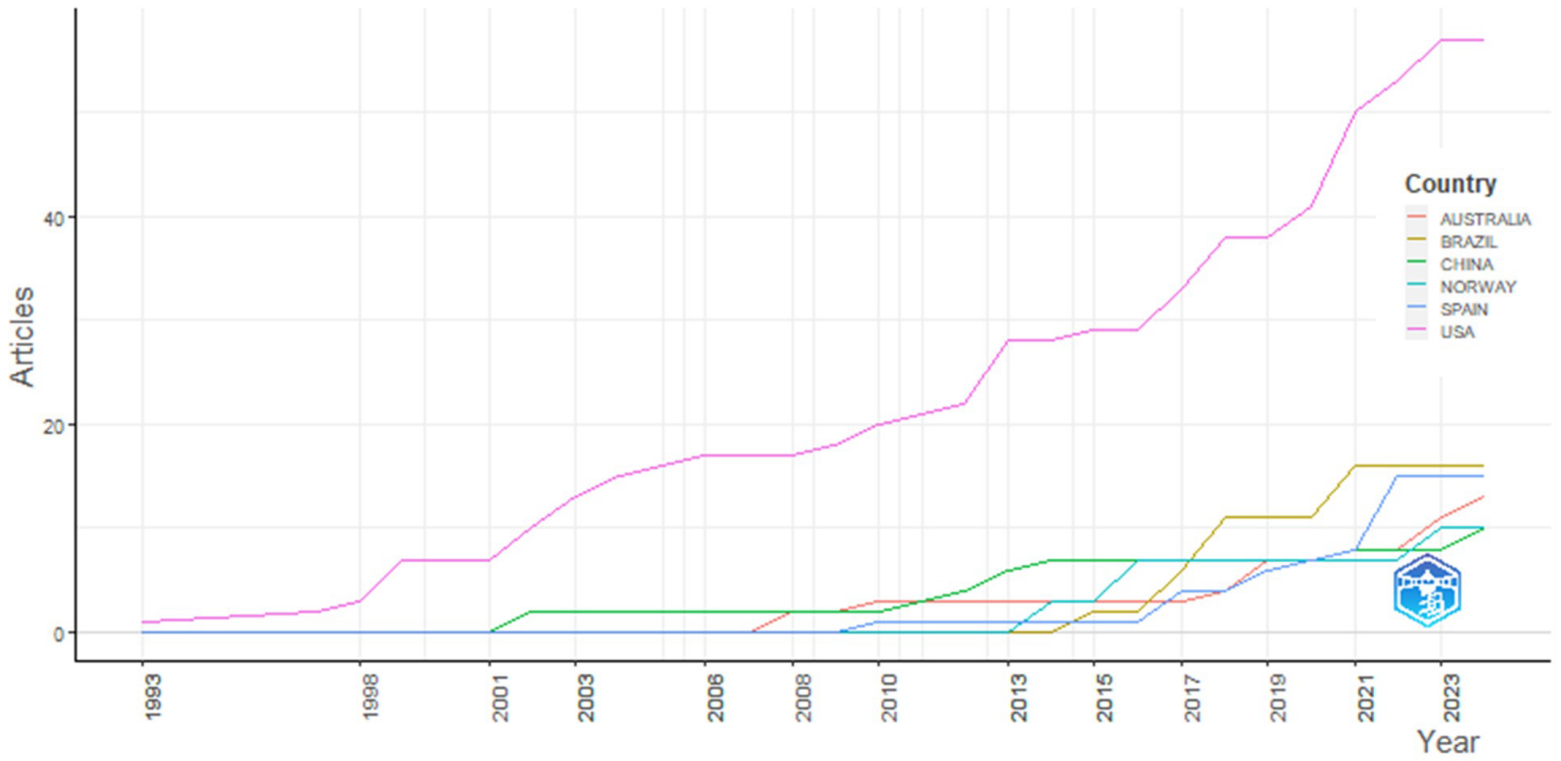
Production by country over time.

Figure 6 shows the publication growth trends in the six most productive countries from 1993 to 2023. Compared with the other five countries, the United States had the most rapidly increasing upward trend in the number of publications since 2001. Since 2007, and especially since 2013, publications in the other five countries have grown steadily, but growth in the United States has been three times higher than it has in the other countries.

**Figure 7:**
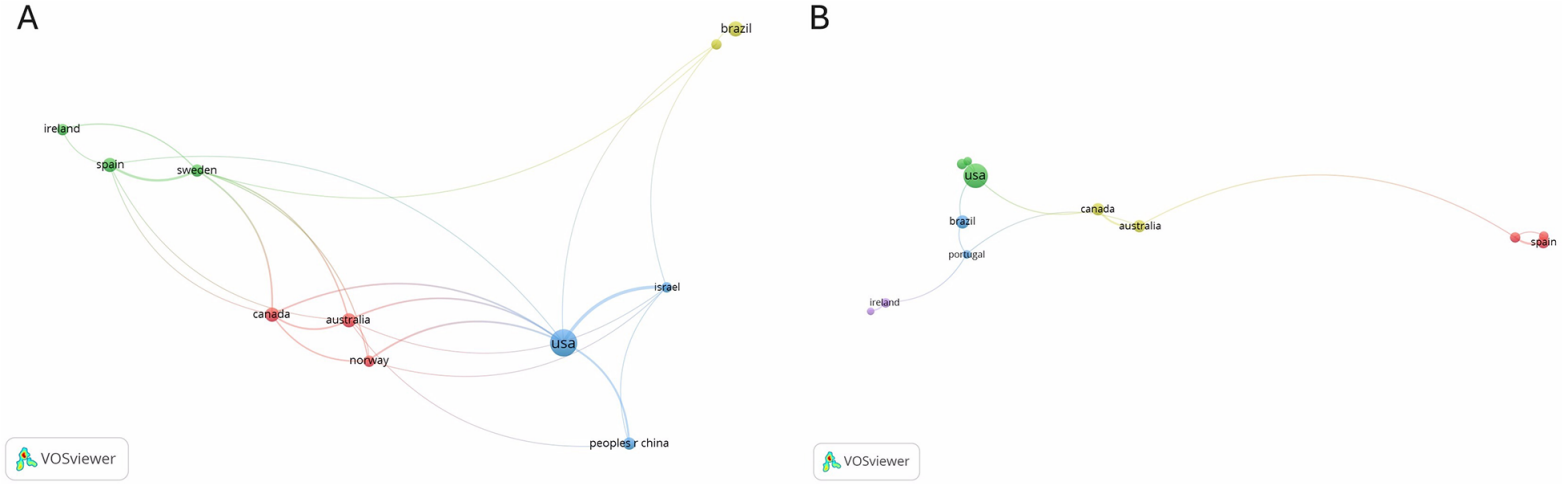
**(A)** citation analysis by country. **(B)** Collaboration networks between countries.

Figures 7A,B show our analyses of citations by country and collaboration networks between countries, conducted using VOSviewer software. The network was constructed with data from 27 countries, and the inclusion criteria were at least four collaborative publications. The analyses resulted in four main citation nodes (Figure 7A) and five collaboration nodes (Figure 7B).

The first citation node (red) grouped together Australia, Canada, and Norway. In this node, Australia had the most active associations, with eight collaborative documents. In addition to Canada and Norway, its main research partners included the United States, Sweden, Spain, Israel, and China.

The second node (green) was centered on Sweden, Spain, and Ireland, with Sweden in the lead with seven collaborative documents. Sweden's main partners were Portugal, Canada, Australia, and Norway.

The third node (blue) revolved around the United States, which produced 29 collaborative documents. Its main connections included China, Israel, Australia, Spain, Canada, Portugal, and Norway.

The fourth node (yellow) focused on Brazil, which had nine collaborative documents. Brazil's main partner in this network was Portugal.

The citation nodes largely aligned with the collaboration nodes between countries. The only exception was the occurrence of a fifth collaboration node (purple) that included Ireland and Greece.

**Figure 8:**
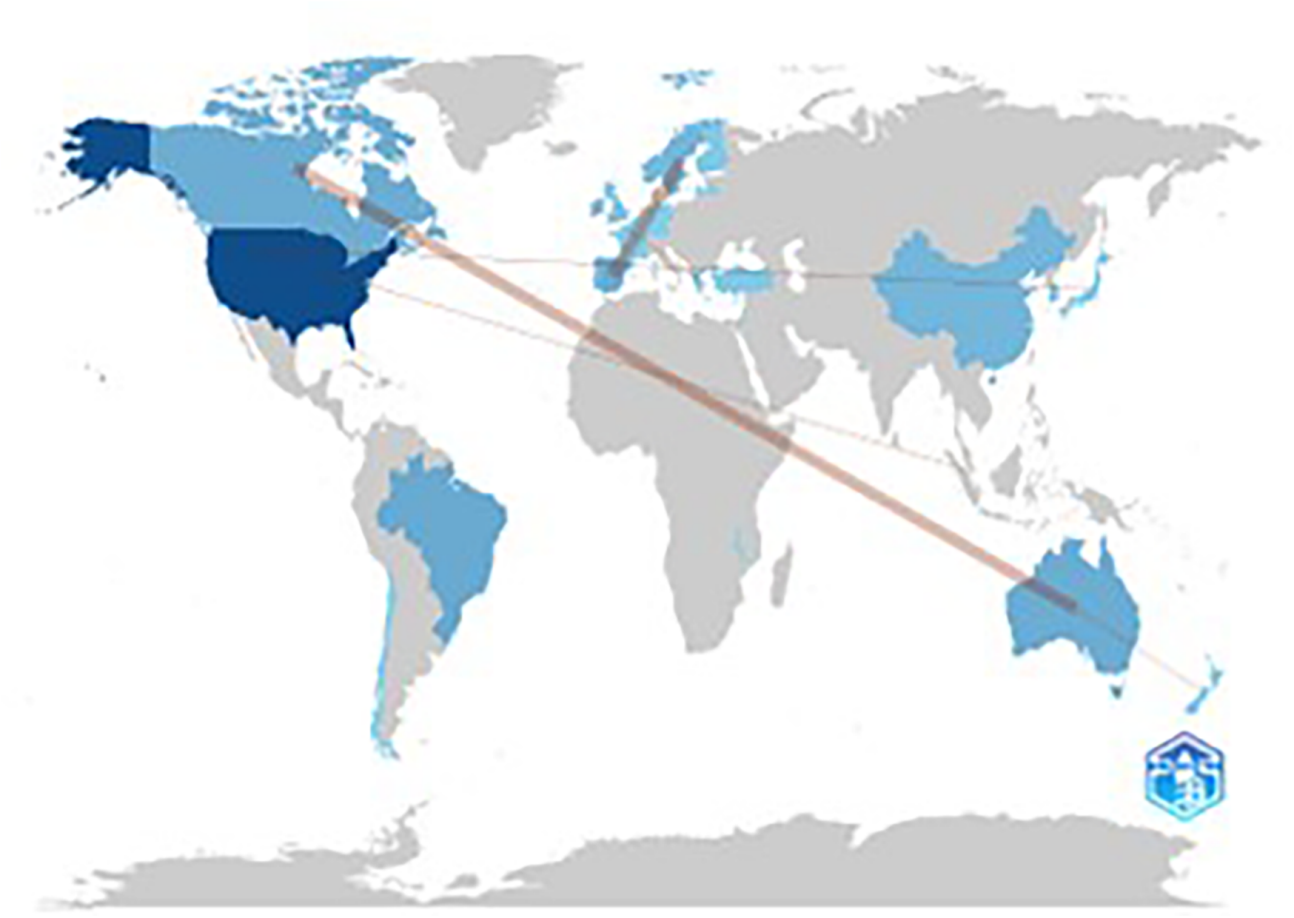
Map of collaboration between countries.

Figure 8 shows the geographic distribution of collaboration between countries during the overall study period. The analysis was conducted using Bibliometrix software and highlighted the strong level of cooperation between Australia and Canada, which had three documents, and New Zealand, which had two. Similarly, Sweden and Spain demonstrated a strong cooperation trend, with three articles.

### 3.4 Key journals and articles

The 83 analyzed publications originated from 142 institutions, according to the available affiliation data. A single article may have multiple authors, and an author may be affiliated with more than one institution. The number of institutions per article ranged from 1 to 9, with an average of 2.6.

Of the total institutions, 71.1% (*n* = 101) supported a single publication, 14.8% (*n* = 21) supported two publications, and 12% (*n* = 17) supported 3–5 publications. Only three institutions (2.1%) supported 7–10 publications.

**Figure 9:**
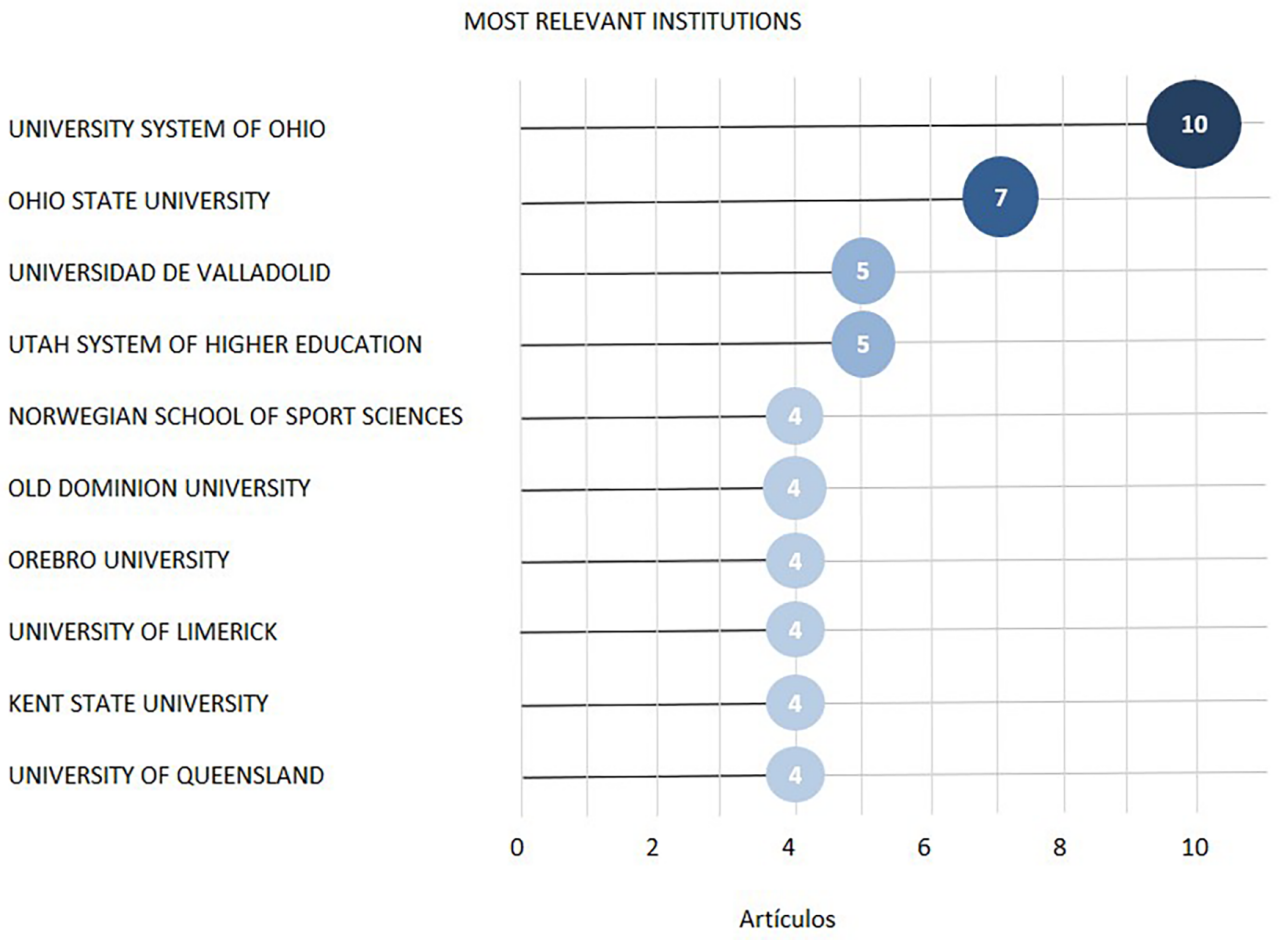
Most relevant institutions according to the number of articles published. (Authors’ creation).

Figure 9 shows the 10 most productive institutions, and the University System of Ohio was the most prolific, with 10 publications. Of these 10 institutions, 5 were located in the United States, while 4 were in Europe (Spain, Norway, Sweden, and Ireland), and 1 was in Australia. Among them, the University of Limerick and Örebro University had the highest average citations per article, with 54.25 and 54.5, respectively.

The relationship between the total citation number and the number of institutions involved in each study revealed a negative correlation, with a coefficient of determination equal to 0, suggesting that these variables were independent (see Table 3).

**Table 3:** The 10 institutions with the highest citation numbers per document.

Institution	Country	Np	Nc	Na	H index
University System Of Ohio	United States	10	352	35.2	9
Ohio State University	United States	7	283	40.43	6
Universidad De Valladolid	Spain	5	235	47	4
Utah System of Higher Education	United States	5	52	10.4	3
Norwegian School of Sport Sciences	Norway	4	62	15.5	4
Old Dominion University	United States	4	71	17.75	3
Orebro University	Sweden	4	210	52.5	3
University of Limerick	Ireland	4	217	54.25	3
Kent State University	United States	4	79	23.25	4
University of Queensland	Australia	4	113	28.25	4

Np, number of publications; Nc, number of citations; Na, average number of citations per article.

While most of the participating institutions were universities, the analysis also revealed the involvement of research centers and institutes as well as some private institutions (see Table 3).

**Figure 10:**
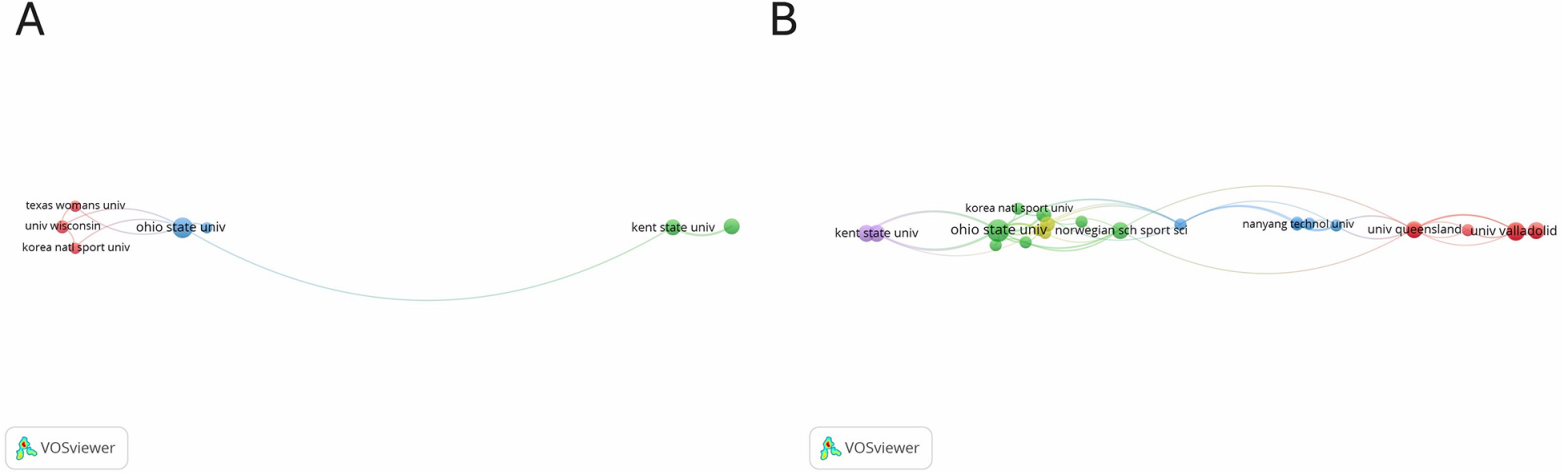
**(A)** institutional collaboration networks. **(B)** Institutional citation networks.

The collaboration network analysis (see Figure 10A) was based upon a minimum of two collaborations between institutions. Of the institutions analyzed, 29 exceeded this threshold, forming three cooperation nodes. The first node (red) highlighted collaborations between the University of Wisconsin, Texas Woman's University, and Korea National Sport University, with three shared documents. The second node (green) involved Kent State University and Old Dominion University, with four documents. The third node (blue) illustrated the strong cooperation between Ohio State University and other institutions, with seven shared documents.

For the citation network analysis (see Figure 10B), the same criterion of a minimum of two collaborations was applied to identify five citation nodes among the 29 institutions that met the threshold. The first node, involving seven institutions, was led by the University of Valladolid and Örebro University, with a link strength of 6 and a TLS of 11, sharing five documents. These institutions formed a citation network with Douglas College, the University of Queensland, the University of Limerick, and the University of Auckland.

The second citation node (green) also included seven institutions, with Ohio State University being the most active, with a link strength of 11, a TLS of 40, and seven shared documents. This university collaborated closely with Georgia State University, Korea National Sport University, the Norwegian School of Sport Sciences, the University of Alberta, and the University of Utah.

The third node (blue) focused on the Chinese University of Hong Kong, which collaborated with Nanyang Technological University, Queensland University of Technology, the University of Jyväskylä, and the University of New Hampshire, although this network was weaker than the others, with only two shared documents.

Finally, the fourth (yellow) and fifth (violet) nodes involved three and two institutions, respectively, with Wisconsin University and Kent State University serving as the main citation hub.

Table 4 shows the 10 journals that published the greatest numbers of articles. Physical Education and Sport Pedagogy was the most productive journal (*n* = 11), followed by Movimento (*n* = 9). In a third-place tie were Adapted Physical Activity Quarterly and European Physical Education Review, each with eight articles.

**Table 4:** The top 10 research journals for physical education practicums.

Journal title	Np	Nc	Na	Impact factor (2023)	Quartile
Physical Education and Sport Pedagogy	11	326	29.64	2.9	Q1
Movimento	9	26	2.89	0.6	Q3
Adapted Physical Activity Quarterly	8	330	41.25	1.7	Q2
European Physical Education Review	8	152	19	2.6	Q1
Sport, Education and Society	7	225	32.14	2.3	Q1
Journal of Teaching in Physical Education	5	57	11.4	1.8	Q2
International Journal of Disability Development and Education	4	171	42.75	1.1	Q3
Teaching and Teacher Education	4	104	26	4.0	Q1
Quest	3	28	9.33	1.6	Q2
European Journal of Teacher Education	2	73	36.5	3.0	Q1

Np, number of publications; Nc, number of citations; Na, average number of citations per article.

Of these journals, 55% published only one article, while the rest (*n* = 13) accounted for 80% of the publications. Among the 10 most productive journals, Adapted Physical Activity Quarterly accumulated the highest citation number (*n* = 330), followed by Physical Education and Sport Pedagogy (*n* = 326).

Of the 29 journals analyzed, 14 were classified Q1 (48.3%), 5 were Q2 (17.2%), 4 were Q3 (13.8%), and another 4 were Q4 (13.8%). The remaining two journals had no impact factor (IF): One was newly created and the other had been removed from WoS. Overall, 65.5% of the studies were published in high-impact journals (Q1 or Q2).

The 29 journals had IFs ranging from 0.7 (Journal of Sport Psychology) to 8.0 (Qualitative Research in Sport, Exercise and Health). We identified 13 journals with IFs ranging from 0.6–2.0, 11 journals with IFs ranging from 2.1–3.9, and 3 with IFs greater than 4.0.

The top 10 journals published 73.5% of the articles and accounted for more than 81.7% of the total citations (1,492 citations). Adapted Physical Activity Quarterly had the highest citation number (*n* = 330), and the average number of citations per article was 22.

**Figure 11:**
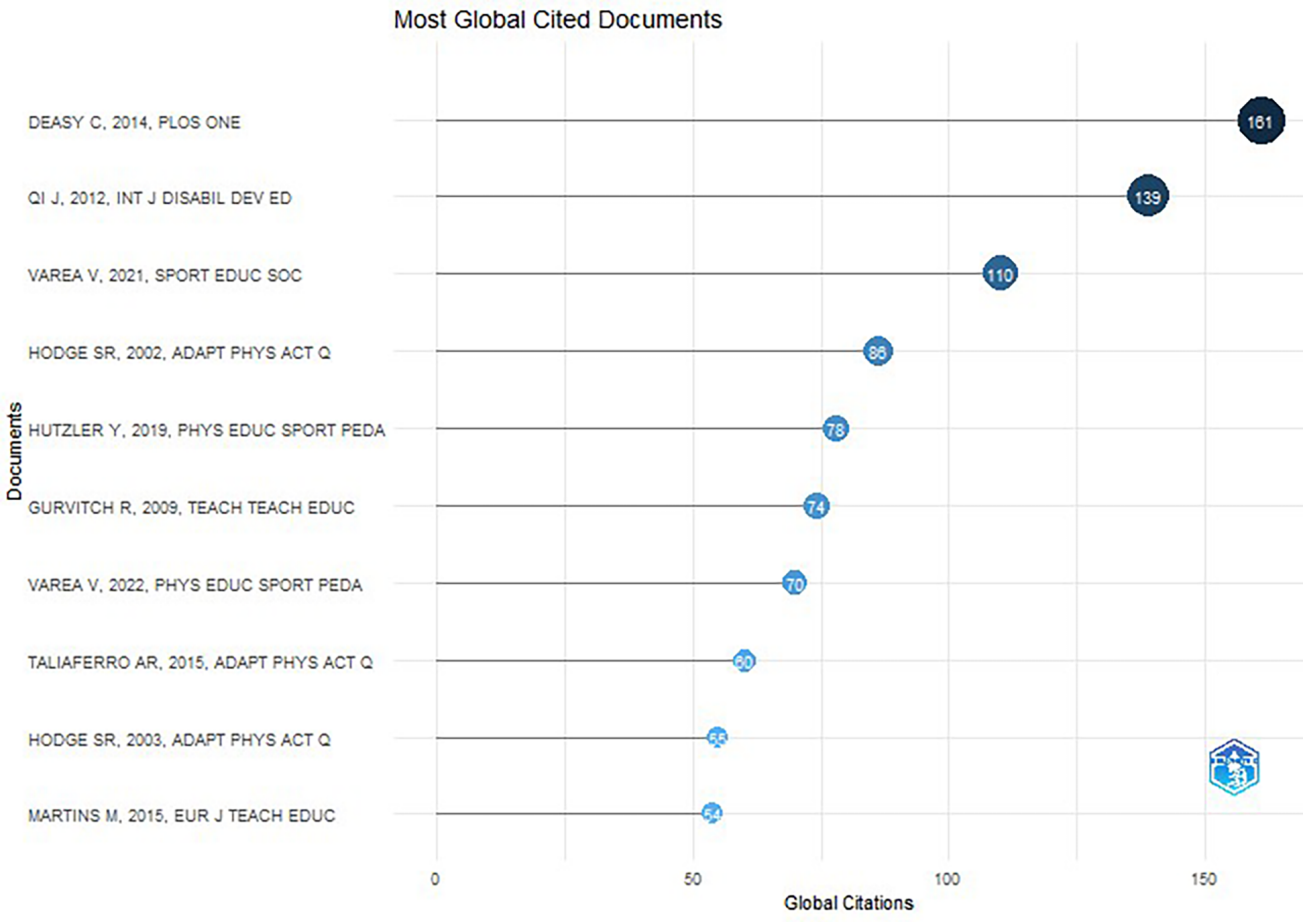
Documents cited most worldwide.

Figure 11 presents the ten most cited publications on Physical Education practicums. The top article was by Deasy et al. (47); it was published in PLOS ONE, and received 161 citations. Titled “Psychological Distress and Coping amongst Higher Education Students: A Mixed Method Enquiry,” this study examines psychological distress among student interns within teacher and nursing education, and how these stressors affect their health and academic performance.

The second most commonly cited article was a review published in 2012 by Qi and Ha titled “Inclusion in Physical Education: A Review of Literature” that received 139 citations. This review analyzed empirical studies on inclusion in Physical Education over the past 20 years and proposed recommendations for future research (48).

The third highest ranked article, by Varea and González-Calvo (49), has been cited 110 times. Titled “Touchless Classes and Absent Bodies: Teaching Physical Education in Times of COVID-19”and published in Sport, Education and Society, this paper explored how the effects of the COVID-19 pandemic have compromised Physical Education practices and the professional development of future Physical Education teachers.

Two studies by Varea and González-Calvo appeared in the top 10 of the most relevant and cited documents, occupying positions 3 and 7. Both works addressed the effects of COVID-19 on students in Physical Education practices. Likewise, the studies by Hodge et al. (2002 and 2003) also appeared in the top 10, at positions 5 and 9; were cited 86 and 55 times, respectively; and were published in Adapted Physical Activity Quarterly.

## 4 Discussion

In recent years, bibliometric studies have grown considerably because they can offer useful indicators on the evolution of science, identify new lines of research, and highlight emerging fields. This study, to the best of our knowledge, represents the first global bibliometric analysis of the literature related to Physical Education practicums, providing an overview of the focal points, themes, and research frontiers in this area.

The data analyzed were collected from 83 articles on practices related to Sports Sciences, specifically in Physical Education, published in the SSCI and SCIE databases. Using tools such as VOSviewer and the R Bibliometrix package (version 4.2.2), this study revealed an increasing trend in the number of annual publications, with a notable increase starting in 2010, and with the highest output occurring in 2018 and 2022. Previous research, such as that by Moy and Rossi (15), has emphasized the growing interest in the formative practices of future Physical Education teachers, suggesting that the scientific output on this topic has yet to mature, which suggests sustained growth in the upcoming years. Similarly, the study by Westerlund (50) suggested that further research in this area is necessary. Likewise, it highlighted the need to better understand the meaning of teaching Physical Education as a practical activity and how the meaning of teaching Physical Education as didactic knowledge is continuously established during the practicum. The keyword co-occurrence analysis revealed three predominant themes of interest—“attitudes,” “Physical Education,” and “student teachers”—which were the key points that attracted the attention of the research community.

The co-authorship analysis revealed the involvement of 198 authors, with an average of 2.95 authors per article, indicating a relatively low level of collaboration compared to similar studies (29, 32). As noted by Mattsson et al. (51), this type of analysis does not always clearly identify individual contributions in collaborative works. Traditionally, the first author is considered the primary contributor, while the last tends to take on a supervisory role (52).

Regarding academic productivity, the h index was uniformly distributed among the most prominent authors, in line with the size of the scientific subcommunities that supported a study's citation number (43). This trend highlights the importance of continuing to foster scientific collaboration, especially in a context in which interdisciplinary and international work is increasingly important (53) and highly valued by scientific evaluation agencies (54).

At the institutional level, the analysis revealed the lack of a solid collaboration network among the field's authors. This fragmentation could be explained by the diversity of educational systems and national legislation types, as local contexts may prevent studies from being comparable or from being able to generalize results globally (50). Nevertheless, some countries—such as Australia, Sweden, the United States, and Brazil—exhibited extensive international cooperation networks, which underscores the importance of collaboration in scientific research (55–57).

In terms of the geographic distribution of the scientific output, advanced economies such as China, North America, and Western Europe led in the publication number. Undoubtedly, these regions can considerably support scientific production because they have strongly developed economies and access to the most innovative and advanced research (58). However, Brazil was the only Latin American country among the top ten producers, possibly due to its greater investment efforts in education (17). Other studies have identified similar patterns regarding the limited participation of regions such as Africa and Latin America in global production, a trend linked to factors such as lower levels of research funding and the lack of institutional development (59, 60).

The analysis of the journals with the highest publication number confirmed that those specializing in Physical Education pedagogy—such as Physical Education and Sport Pedagogy, Movimento, and European Physical Education Review—accounted for a large portion of the work in this field. These results are consistent with Bradford's Law, which explains the concentration of scientific productivity in a small core of journals (61). Publishing in high-impact journals offers numerous advantages, such as recognition in the scientific community and career advancement (62, 63).

Finally, recent changes in journal classification by Clarivate Analytics, particularly the extension of IFs to journals in the Arts and Humanities Citation Index and the Emerging Sources Citation Index, may influence the perception of impact indices and quartiles. This development presents new challenges for evaluating publication quality and conducting bibliometric analyses (64).

## 5 Conclusions

To the best of our knowledge, this is the first bibliometric study to analyze Physical Education practicums on a global scale. The results reveal considerable growth in research on this topic, with a noticeable increase in publications starting in 2010. Three predominant themes were identified: “attitudes,” “Physical Education,” and “student teachers.” However, low collaboration among authors was observed, possibly due to the global fragmentation of educational systems and differences in national legislation. Countries like Australia, Sweden, the United States, and Brazil demonstrate strong international cooperation networks. Geographically, advanced economies dominate scientific output, while regions such as Africa and Latin America have limited participation, likely due to lower funding and institutional development. Finally, journals specializing in Physical Education pedagogy account for most of the publications, following Bradford's Law. Recent changes in journal classification may influence the perception of impact indices and publication quality.

## Data Availability

The original contributions presented in the study are included in the article/Supplementary Material, further inquiries can be directed to the corresponding author.
